# Facing stereotypes: ERP responses to male and female faces after gender-stereotyped statements

**DOI:** 10.1093/scan/nsaa117

**Published:** 2020-09-09

**Authors:** Pablo Rodríguez-Gómez, Verónica Romero-Ferreiro, Miguel A Pozo, José Antonio Hinojosa, Eva M Moreno

**Affiliations:** Human Brain Mapping Unit, Instituto Pluridisciplinar, Universidad Complutense de Madrid, Madrid, Spain; Department of Experimental Psychology, Cognitive Processes and Speech Therapy, Universidad Complutense de Madrid, Spain; Human Brain Mapping Unit, Instituto Pluridisciplinar, Universidad Complutense de Madrid, Madrid, Spain; Biomedical Research Center in Mental Health Network CIBERSAM, Spain; Human Brain Mapping Unit, Instituto Pluridisciplinar, Universidad Complutense de Madrid, Madrid, Spain; Human Brain Mapping Unit, Instituto Pluridisciplinar, Universidad Complutense de Madrid, Madrid, Spain; Department of Experimental Psychology, Cognitive Processes and Speech Therapy, Universidad Complutense de Madrid, Spain; Languages and Education Department, Universidad de Nebrija, Madrid, Spain; Human Brain Mapping Unit, Instituto Pluridisciplinar, Universidad Complutense de Madrid, Madrid, Spain; Biomedical Research Center in Mental Health Network CIBERSAM, Spain

**Keywords:** event-related potentials (ERPs), face processing, gender stereotypes, N170, LPP

## Abstract

Despite gender is a salient feature in face recognition, the question of whether stereotyping modulates face processing remains unexplored. Event-related potentials from 40 participants (20 female) was recorded as male and female faces matched or mismatched previous gender-stereotyped statements and were compared with those elicited by faces preceded by gender-unbiased statements. We conducted linear mixed-effects models to account for possible random effects from both participants and the strength of the gender bias. The amplitude of the N170 to faces was larger following stereotyped relative to gender-unbiased statements in both male and female participants, although the effect was larger for males. This result reveals that stereotyping exerts an early effect in face processing and that the impact is higher in men. In later time windows, male faces after female-stereotyped statements elicited large late positivity potential (LPP) responses in both men and women, indicating that the violation of male stereotypes induces a post-perceptual reevaluation of a salient or conflicting event. Besides, the largest LPP amplitude in women was elicited when they encountered a female face after a female-stereotyped statement. The later result is discussed from the perspective of recent claims on the evolution of women self-identification with traditionally held female roles.

## Introduction

Stereotypes in general reflect expectations about members of a particular social group. Specifically, gender stereotypes relate to those characteristics, preferences and ambitions of women and men and that may or may not fit actual social group differences. Contemporary social interactions are often influenced by stereotypes of masculine roles traditionally linked to professional achievement and competition and feminine roles of empathy and family member care-givers (Nosek *et al.*, 2002). Since gender is a primary feature in other’s recognition, its immediacy, salience and easiness to polarize, contribute to their formation and persistence (Ellemers, 2018). However, gender stereotypes are also dynamic and prone to be shaped by human evolution and societal changes (Zafra and Garcia-Retamero, 2011). In recent years, women have begun to adopt roles traditionally held by men and to develop novel feminine effective roles, although the opposite tendency shows a slower trend (Diekman and Eagly, 2000; Vandello *et al.*, 2013). Additionally, the results from recent studies show that they do not entirely embrace the stereotypic view of women (Hentschel *et al.*, 2019). The question is how deep stereotypical beliefs about men and women are rooted in human brain cognition, how malleable they can be, and if these beliefs are indeed changing in contemporary times.

To answer these questions, event-related potentials (ERPs) become a very useful tool as it provides a direct online measure of spontaneous brain activity linked to the processing of stimuli even in the absence of any explicit task (e.g. a gender categorization task).

At the present, some language comprehension ERP studies have shown that gender stereotype violations indeed modulate brain activity (Osterhout and Holcomb, 1992; Van Berkum *et al.*, 2008; White *et al.*, 2009; Canal *et al.*, 2015; Proverbio *et al.*, 2017, 2018a). In these studies, ERP responses are typically time-locked to a target word (a personal or reflexive pronoun or a noun) that makes a sentence relatively easier/harder to process according to previously held stereotyped occupational or societal roles for men and women (e.g. ‘The doctor/nurse prepared “himself/herself” for the operation’; ‘Prepared the tomato sauce and then “shaved”’). These studies using exclusively linguistic material reveal changes in the amplitude of two ERP components, the N400 and P600, which are sensitive to word expectancy in context (N400) (Kutas and Federmeier, 2011) and sentence syntactic complexity or ambiguity (P600) (Osterhout and Holcomb, 1992; Niharika and Prema Rao, 2020).

For example, modulations in the N400 ERP component were observed by Van Berkum *et al.* (2008). In their study, participants heard well-formed sentences, such as ‘Before I leave I always check whether my make-up is still OK’, spoken in a female (congruent trial) or a male (incongruent trial) voice. Speaker inconsistencies elicited larger N400 amplitudes relative to congruent trials. Likewise, White *et al.* (2009) found N400 amplitude increases to stereotypical incongruent relative to congruent prime-target word pairs (e.g. Men—Nurturing; Women—Nurturing). In a recent study by Proverbio *et al.* (2018a), only male participants showed effects of gender prejudices. In addition, the violation of male and female stereotypes for them led to qualitatively distinct ERP effects (an N400 effect for unexpected male agents in a female role, and P600 modulations for unexpected female agents in a male prejudiced sentence). The authors concluded that the degree of self-identification with a woman or a man agent might be critical for this asymmetry.

With regard to studies in which P600 modulations were mostly found, Osterhout *et al.* (1997) asked participants to read sentences where the subject’s gender could be semantically established (‘mother, king’) or stereotypically biased by using occupational professions (‘nurse, mechanic’). The violation of the latter elicited enhanced P600 amplitudes, particularly larger in female participants. Canal *et al.* (2015) also found modulations in the P600 amplitude in response to reflexive pronouns that clashed with gender stereotypes (‘engineer’). Interestingly, the authors performed linear mixed models analyses using the scores on three different scales evaluating sexist attitudes. They claim that the way to link an anaphor with the antecedent was personal-trait dependent. Particularly, those participants with lower scores in the femininity index of the Bem Sex-Role Inventory (BSRI-f) and the Ambivalent Sexism Inventory (ASIh), seemed to actively engage in searching for an appropriate although less likely antecedent (indexed by a Nref rather than a P600 effect) (Van Berkum *et al.*, 2008, 2009). This result highlights the importance of considering individual differences when investigating gender stereotype effects.

In contrast to words, faces are a special class of stimuli due to its particular biological and social significance (Carey, 1992). They are detected faster (Bruce and Young, 1986; Nachson, 1995) and are more likely to be perceived under conditions of inattention or divided attention (Mack and Rock, 1998) compared with other stimuli. Although faces have usually been studied in isolation, in the absence of any contextual reference, some studies have examined how face processing is influenced by the context in which they are presented (Abdel Rahman, 2011; Wieser and Brosch, 2012; Wieser *et al.*, 2014; Kissler and Strehlow, 2017; Baum *et al.*, 2020). A behavioral study conducted by Righart and de Gelder (2008) reported faster recognition for emotional expressions presented on emotionally congruent contexts, when compared to those faces presented on incongruent contexts. In the electrophysiological domain, the N170 is the earliest ERP component identified in most studies as being sensitive to faces (Bentin *et al.*, 1996). Its latency ranges between 140 and 200 ms and has an occipitotemporal scalp distribution (see reviews by Rossion, 2014; Olivares *et al.*, 2015). This component has been related to the stage of structural coding proposed by traditional models of face perception (Bruce and Young, 1986), a stage where visual representations of features of individual faces are generated, such as gender or age. Face recognition (familiarity) occurs at a later stage (250–500 ms) (Bentin and Deouell, 2000). Nonetheless, modulations at the early N170 component were found when faces were embedded in situational contexts. Particularly, emotionally biased sentences followed by emotionally incongruent faces enhanced the amplitude of the N170 (Dieguez-Risco *et al.*, 2015). Increases in N170 amplitude have also been observed as a function of the intensity of emotional facial expressions, regardless of the quality of the emotion (disgust, fear or anger), which was interpreted as a coding for saliency of external stimuli (Sprengelmeyer and Jentzsch, 2006). Moreover, explicit instructions to allocate attention to the facial stimuli increase N170 amplitude (Eimer, 2000; Churches *et al.*, 2010). In fact, a very recent study pointed out that N170 amplitude can be considered an implicit prejudice measure (Giménez-Fernández *et al.*, 2020). In contrast, other studies found no early perceptual ERP effects (i.e. N1 or Early Posterior Negativity, EPN) in response to facial stimuli as a function of whether they followed emotionally negative or positive information (e.g. ‘This cruel kidnapper killed a girl’ *vs* ‘This diligent gardener watered the flowers’) (Kissler and Strehlow, 2017). Late (500–700 ms) frontal and parietal ERP effects were instead obtained, respectively, at the encoding and at the subsequent recognition of the face, indexing post-perceptual reevaluation top-down processes. Nonetheless, in a similar study, an early EPN enhancement was found for negative biographical information for well-known faces (Abdel Rahman, 2011). These authors also found late (400–600 ms) parietal modulations (late positivity potential [LPP]) based on prior affective knowledge in this case for both well-known as well as unfamiliar faces. Thus, contextual information always affected post-perceptual evaluative processes (indexed by the LPP), whereas it only affected earlier perceptual aspects of face processing (EPN) for well-known faces.

Some studies have observed LPP modulations in response to faces in context (Aguado *et al.*, 2013, 2019a, 2019b; Dieguez-Risco *et al.*, 2015). It typically appears between 400 and 700 ms over parietal-occipital electrodes (Cuthbert *et al.*, 2000; Weinberg and Hajcak, 2010; Breton *et al.*, 2014; Thom *et al.*, 2014). This later positivity reflects increased attention to the salience of incongruent stimuli (Hajcak *et al.*, 2010). Those studies in which faces were preceded by sentence contexts have found larger amplitudes for incongruent relative to congruent targets (Aguado *et al.*, 2013 2019a, 2019b; Dieguez-Risco *et al.*, 2015). Social hierarchy, particularly occupational status, a variable that might be intertwined with gender roles, shows a larger LPP to high *vs* low status only in female participants in the study conducted by Breton *et al.* (2019). Also, this component has been found to be sensitive to social expectancy violations, when participants read sentences describing behaviors inconsistent with a previously implied personality trait (Bartholow *et al.*, 2001; Baetens *et al.*, 2011).

To date, ERP variations in the processing of a facial stimulus as a result of a gender stereotype compliance or violation have not yet been examined. Thus, in contrast to previously reviewed linguistic studies that included grammatically marked violations of a stereotypical gender, in the current study, we created grammatical genderless correct sentence contexts (i.e. with omitted personal and gender ambiguous reflexive pronouns) that were followed by the presentation of a male or a female face. These sentence contexts aimed to bias participants’ expectancies toward a specific gender (e.g. ‘Preparó el almuerzo de los niños antes de ir al trabajo’; (He/she, omitted) prepared the kids’ snack before going to work). In Spanish, omitted personal pronouns as well as genderless possessive pronouns (e.g. ‘su’, meaning his or her) are common. With this design, the subject gender was unspecified within the sentence and participants did not know if the statement referred to a man or a woman. Eventually, the face after the sentence solved the ambiguity.

The aim of this design was twofold. First, it allowed us to explore whether early latency ERP components related to face processing (N170) reflect prior expectancies triggered by gender-stereotyped roles, attitudes and behaviors. Changes in the amplitude of this component are expected since previous studies have shown that emotionally mismatching contexts increase its amplitude (Dieguez-Risco *et al.*, 2015). Second, it allowed us to explore later LPP effects, in which the salience of incongruent stimuli becomes critical (Hajcak *et al.*, 2010). In addition, our design has some methodological aspects that were not present in previous studies. First, we were able to introduce a control condition in which male and female faces were preceded by un-stereotyped sentences (e.g. ‘Usa gafas para leer’; ‘(He/she) uses glasses just for reading’). In addition, gender-biased stereotypes referred to a wider range of aspects beyond occupational professions, such as personal traits, preferences, behavior and societal expectancies. The sex of the participant was included as a variable to explore whether men and women might process stereotype violations differentially (Proverbio *et al.*, 2018a). Finally, following the procedure by Canal *et al.* (2015), we use an individual differences approach by performing linear mixed models analyses. Osterhout *et al.* (1997) highlighted the possibility that ‘the amplitude of the positive shift reflects the strength of stereotypic beliefs’. To test this hypothesis, we looked for a co-variation between electrophysiological results and individual scores on the Modern Sexism test (Swim *et al.*, 1995).

Since gender stereotyping is a pervasive phenomenon (Ellemers, 2018) and it is a salient feature in face recognition, we hypothesize that the N170 might be modulated by gender stereotyping, considering that it has been modulated in similar paradigms where faces were preceded by (mis)matching contexts. We expect that faces that do not fit well with the preceding stereotyped statement (a female face after a stereotyped male statement and vice versa) might result in increased N170 amplitudes.

In addition, we predict that a mismatch between a gender-stereotyped sentence and an accompanying opposite sex face would increase LPP responses, indicating that the violation of gender stereotypes incurs in a cost over subsequent face processing stages. Modulations on such late ERP component during face processing will be interpreted as a difficulty of integration of a prediction with the actual stimulus.

Regarding the influence of the sex of the participant, larger gender mismatch effects were found for females in the study carried out by Osterhout *et al*. in the USA in the nineties (1997). In contrast, larger amplitudes in response to stereotype violations were found in men in the rather recent study by Proverbio *et al.* (2018a) with Italian participants. In the latter study, a dissociation also occurred in men’s responses depending on the type of gender stereotype violation, with a different ERP pattern for violations of male and female stereotypes. Our study might potentially also reveal a qualitatively different response (at the level of N170 and/or LLP) for unexpected mismatching male or female faces depending on the sex of the viewer.

## Methods

### Participants

Forty-two Spanish native speakers (22 women and 20 men) participated in the experiment in exchange for class credits. All participants provided informed consent to take part in the study and reported normal or corrected-to-normal vision, and no history of neurological or psychiatric disorders. Data from two female participants were discarded because they did not contribute at least 60% artifact-free trials to one or more of the experimental conditions. Thus, the final sample consisted of 20 women (mean age = 20.5 years, range = 18–35 years, s.d. = 4.02) and 20 men (mean age = 20.75 years, range = 18–31 years, s.d. = 3.24). No differences in terms of age were found (t = 0.433, *P* = 0.668). From this sample, 33 participants reported being right-handed. The average handedness score (Oldfield, 1971) was + 56.51 (range = − 100 to + 100).

### Stimuli

**Fig. 1.**
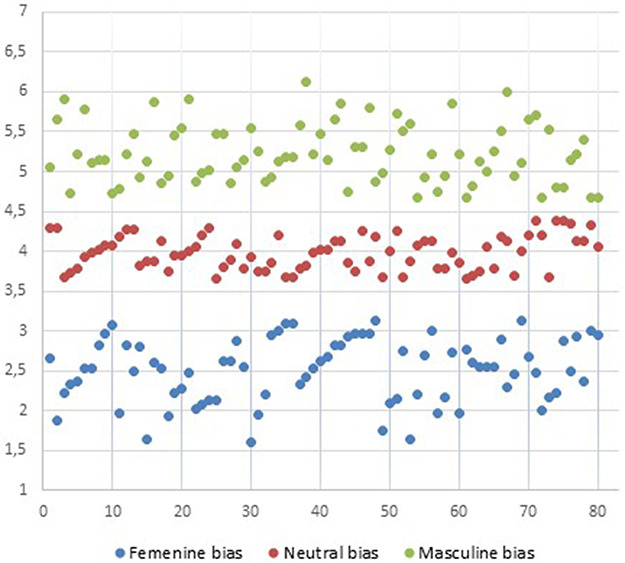
Score bias distribution for feminine-biased sentences, masculine-biased sentences and neutral sentences. Values closer to 1 corresponds to ‘I’m sure this sentence refers to a woman’ and closer to 7 corresponds to ‘I’m sure this sentence refers to a man’.

An initial set of experimental stimuli was created. It consisted of 300 sentences, which described roughly the personality, interests and societal expectations of a certain individual. The gender of the subject was never specified in any of the sentences. Sentences were divided in three groups (100 per group): (i) Female-gender-biased; (ii) Male-gender-biased and (iii) Neutral sentences, which did not bias toward any particular gender. Some examples are shown in Table 1. This set of stimuli was subjected to a norming study with 80 participants (40 women and 40 men, none of whom participated in the following ERP study). Participants were told to evaluate these sentences using a 7-point Likert scale (ranging from 1 ‘I’m sure this sentence refers to a woman’ to 72, ‘I’m sure this sentence refers to a man’). Participants were instructed to avoid making any evaluative/moral judgment and just answer to this scale honestly. The final set consisted of 80 female-gender-biased sentences (mean = 2.50, range [1.60–3.12]; s.d. = 0.42), 80 neutral sentences (mean = 3.98, range [3.65–4.37]; s.d. = 0.29) and 80 male-gender-biased sentences (mean = 5.22, range [4.67–6.12]; s.d. = 0.49). Figure 1 represents the score distribution of the final set of sentences.

**Table 1.** Examples of the sentences used in the experiment

	Sentence	Translation
Feminine bias	Fue a una entrevista de trabajo y le preguntaron si pensaba tener hijos. Su juguete favorito de la infancia era una Barbie. Cada semana estrena un modelito nuevo. Dijo que tenía menos años que su verdadera edad.	(He/she) was asked at a job interview if (he/she) had plans to have babies. His/her favorite toy as a child was a Barbie doll. (He/she) wears a new outfit every week. (He/she) lied about her age saying (he/she) was a year younger.
Gender unbiased	Duerme 8 horas al día. Habla perfectamente dos idiomas. Usa gafas solamente para leer. El año pasado se trasladó a Madrid para hacer un máster.	(He/she) sleeps 8 hours per day. (He/she) fluently speaks two languages. (He/she) wears glasses just for reading. (He/she) moved to Madrid last year to enroll on a master’s degree.
Masculine bias	No tiene miedo al volver a casa por la noche. En el restaurante pidió un chuletón de 750 g. Se fumó un puro en la boda después de comer. Se sienta en el metro con las piernas abiertas.	(He/she) is not scared when coming back home at night. (He/she) asked for a 750 g piece of meat at a restaurant. (He/she) smoked a cigar at the wedding. (He/she) spreads his legs when sitting in the subway.

In addition, 80 black and white photographs of Caucasian models showing a neutral expression (40 female faces and 40 male faces) were used during the experimental procedure. Fifteen of these photographs were taken from the NimStim database (Tottenham *et al.*, 2009), and the rest was taken from KDEF database (Lundqvist *et al.*, 1998). Photographs were equated in terms of luminance, size and contrast. The faces were presented centered on the screen, inside a 13 × 10 cm square, subtending an area of 11.42 × 8.79º of visual angle.

### Procedure

Participants were fitted with encephalogram (EEG) electrodes while they filled out handedness, vision and health questionnaires. They were seated approximately 65 cm in front of a 19" computer monitor. The task was presented using the Psychtoolbox software package (Brainard, 1997; Kleiner *et al.*, 2007), a toolbox implemented in Matlab environment (The MathWorks, Natick, MA).

**Fig. 2.**
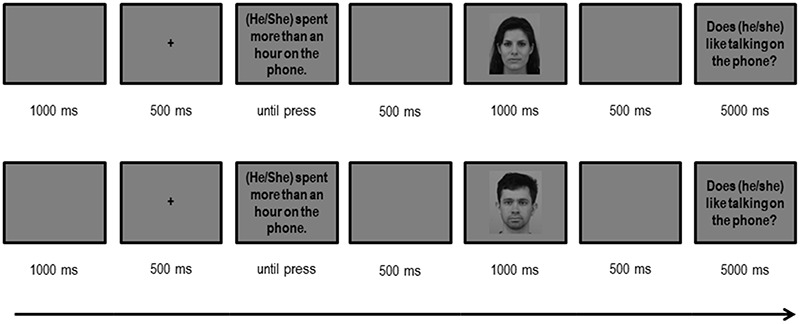
Two examples of a trial sequence with male and female faces.

The session began with a short set of practice stimuli to acclimate the participants to the silently reading task. The gender-biased sentence was presented on the screen until the participants pressed the spacebar. All words in the sentence were presented in a black 40-point lower-case Arial font on a grey background. After an interval of 500 ms, a face was presented in the center of the screen for a second duration. Participants could encounter a congruence between the gender-biased sentence, an incongruence or simply a face after a gender-unbiased sentence as a control condition. The ERP response was time-locked to the presentation of the faces. Once the face disappeared, in 20% of the trials (48 trials) participants encountered a ‘yes/no’ comprehension question related to the previous sentence. These questions were used in order to keep participants in a high-attention state throughout the task and were shown in a blue 40-point lower case Arial font. Response buttons were counterbalanced across participants. Once the question was answered, a new trial began after 1000 ms; Figure 2 schematizes the structure of a whole trial.

Sentences were presented in random order and divided into four blocks, with a break between them. Each face was presented three times in three different conditions (congruence sentence-face; incongruence sentence-face; control condition). Break’s duration was unlimited; participants decided when to start the next block. The whole session lasted about 40 minutes. At the end of the session, participants filled out the Modern Sexism test (Swim *et al.*, 1995), a short test to study the increasingly subtle and covert prejudice and discrimination against women (Benokraitis, 1986). It is a short test consisting of 8 items. Those high in Modern Sexism show less sympathetic responses toward women’s issues and are more likely to perceive greater equality than actually exists.

### EEG data recording and preprocessing

EEG data were recorded from 64 Ag/AgCl electrodes distributed according to the 10 to 20 international system (‘American Electroencephalographic Society guidelines for standard electrode position nomenclature’ 1991). These electrodes were mounted in an electrode cap (Electro-Cap International) and their impedances were kept below 5 kΩ. Electrodes were referenced online to the left mastoid and amplified with a Brain Amps amplifier at a sampling rate of 1000 Hz. The signal was filtered through a 0.1–100 Hz online band-pass filter. The electrooculographic activity was recorded using vertical and horizontal bipolar electrodes placed at a supra-infraorbital level of the right eye and on the outer canthus of both eyes, respectively.

Data was processed using BrainVision Analyzer software (Brain Products, Munich), re-referenced off-line to the mastoids average for the LPP component, and to the average cephalic reference for the N170 component, following the standard procedure for N170 analysis (Wang *et al.*, 2019). Bipolar horizontal and vertical electrooculograms (EOGs) were corrected using Gratton *et al.* method (Gratton *et al.*, 1983). For artifact rejection purposes, the following thresholds were set: maximal allowed voltage step, 50 µV; minimal and maximal allowed amplitude, ± 100 µV; lowest allowed activity (max-min), 5 µV for an interval length of 1500 ms. Once any threshold was met in the continuous EEG file, data recorded at that point were marked and discarded, together with data recorded during the 200 ms before and after the detection. This was performed to avoid including any residual artifacts in subsequent computations of ERP averages. EEG raw data from all subjects were scanned and marked using the same criteria. For the 40 participants, 7.98% of trials were discarded and an average of 37.3 trials remained per experimental condition. A Butterworth zero phase filter was applied to the EEG data (low cutoff at 0.1 Hz, time constant = 1.6 s, 24 db/oct; high cutoff at 20 Hz, 24 dB/oct). The high cutoff filter at 20 Hz was used to smooth high frequency activity present in the EEG The continuous EEG was segmented into 1000 ms epochs starting 100 ms before the onset of the target face. Artifact-free average waveforms were then computed for each condition separately, after subtraction of the pre-stimulus baseline.

### ERP analysis

Analyses of mean amplitudes in regions of interest (ROIs) were conducted using linear mixed-effects models via maximum likelihood estimation as we only have one random effect and data are balanced. All analyses were conducted with the lme4 package and the function lme4::lmer() (Bates *et al.*, 2015), in the R language for statistical computing (R Core Team, 2014).

Analyses were performed on the average voltage within pre-defined spatiotemporal ROIs for each trial following standard procedures in previous face processing studies (Cuthbert *et al.*, 2000; Weinberg and Hajcak, 2010; Breton *et al.*, 2014; Thom *et al.*, 2014; Valdes-Conroy *et al.*, 2014). Given that some studies have shown differences in the lateralization of the N170 for men and women two spatial ROIs were pre-defined:: P7 and PO7 electrodes (left hemisphere), and P8 and PO8 electrodes (right hemisphere) for the N170 component; and a single spatial ROI for the LPP component, including electrodes: P1, PZ, P2, PO3, POZ, PO4 (Proverbio *et al.*, 2012; Godard *et al.*, 2013). Activity per trial was averaged within a time window of 165 to 175 ms after face onset for the N170 component, and within a time window of 400 to 700 ms after face onset for the LPP component.

The model is made up of two major components: the fixed effects and the random effects. Variance across participants was modeled as random intercept terms in the statistical model. Predictors of amplitude variance included the participant’s sex, the bias of the sentence, the gender of the face (discrete variables), the sentence bias score, and the Modern Sexism test score (continuous variables). Fixed-effect parameter estimates should be interpreted as the regression weights in the linear regression model (Cohen *et al.*, 2003; Andrew, 2017). Higher-order interactions between the participant’s sex, the sentence bias and the gender of the face were analyzed, following our theoretical interests.

This type of analysis allows us to examine the impact of the participant’s sex, the sentence bias, the gender of the face and their interaction on the different brainwave components (N170 and LPP). Additionally, we included the Hemisphere factor in the N170 analyses in order to explore gender differences in lateralization.

Non-significant variables were not included in successive models to obtain a final adjusted model that explains our dependent variable.

Regarding continuous variables, parameter estimates reflect change in mean amplitude per standard deviation change in the variable. For discrete variables, effect sizes reflect change in mean amplitude between the reference and contrast group. Parameter estimates for higher-order interactions reflect the magnitude of the effect of one of the independent variables on a dependent variable as a function of two (or more) independent variables.

Effects sizes are presented as model-derived fixed-effect parameter estimates along with corresponding 95% profile likelihood confidence intervals (CIs) for statistical inference (Cumming, 2014). Parameters with CIs that do not contain zero are interpreted as statistically significant following traditional null-hypothesis significance testing.

## Results

### Modern sexism test

Scores obtained by men were only slightly higher (mean = 17.6, s.d. = 4.71, range = 8–28) than for women (mean = 15.75, s.d. = 4.84, range = 8–28). However, the difference in mean scores for men and women were not statistically significant (*t*(38)* *= 1.22, *P* = 0.23).

### N170

**Fig. 3.**
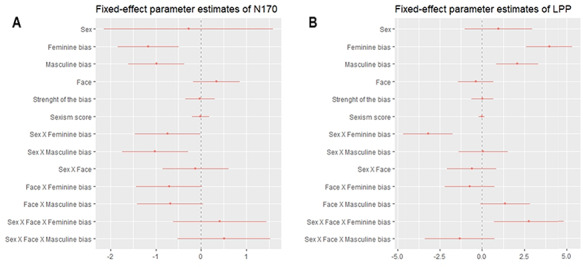
Fixed-effect parameter estimates and corresponding 95% profile confidence intervals of N170 (3A) and LPP (3B). Estimates with intervals containing 0 do not meet traditional levels of statistical significance.

As stated previously, non-significant variables were subsequently removed from the complete model until reaching a reduced model including all the statistically significant variables and its interactions. The fixed-effects parameter estimates and their corresponding 95% CIs from the linear mixed effects of the complete model of N170 component are presented in Figure 3A. According to these results, the reduced model finally obtained is specified as follows:

\begin{align*} & {\rm{N170} \lt-\rm{lmer}} \left( {{\rm{Voltage} \sim \rm{Sex + Bias + Hemisphere+ Sex^{*}Bias }}}\right. \nonumber\\ &\hspace*{-2pt}+ \left.{{{\rm Sex^{*}Hemisphere + }}\left( {{\rm{1|Subject}}} \right)\!, {\rm{data = Peaks\_N170, REML}}}\right. \nonumber\\ &\hspace*{-2pt}= \left.{{{\rm FALSE}}} \right)\end{align*}

**Fig. 4.**
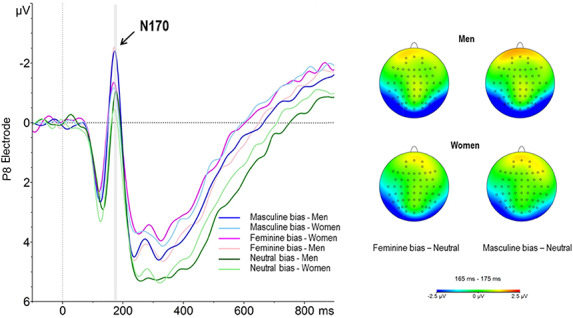
ERP responses elicited by gender-stereotyped and neutral sentences in men and women. Responses are plotted at P8 electrode. Negative voltage is plotted up (left panel). Voltage scalp distributions (right panel).

Both feminine and masculine bias of the sentence were significant (b = − 1.55, 95% CI = [−1.92; − 1.19]; b = − 1.35, 95% CI = [−1.71; − 0.98], for feminine and masculine respectively). Furthermore, the interaction of Sentence bias × Sex of the participant was also significant (b = − 0.53, 95% CI = [−1.04; − 0.01]; b = − 0.76, 95% CI = [−1.27; − 0.25], for feminine and masculine bias respectively). Grand-average ERPs illustrating both first-order interactions are shown in Figure 4. The difference between the neutral bias with respect to feminine and masculine bias is larger in male participants compared with females. Specifically, collapsing across the gender of the face, the N170 amplitude was larger when male participants were presented with feminine or masculine biased sentences, compared with neutrally biased sentences. The same effect was found in female participants to a lesser extent. Finally, the interaction of Sex × Hemisphere was also significant (b = 0.44, 95% CI = [0.08; 0.81]). Post hoc analyses showed significant inter hemispheric differences in both male and female participants, being the magnitude of such differences larger for female (1.15 µV, *P* = < 0.001) than for male (0.7 µV, *P *= < 0.001). In both cases, N170 amplitudes indicated a right hemisphere dominance.

### LPP

The plot of parameter estimates corresponding to the complete model is presented in Figure 3B. Then, the reduced model resulting from removing the non-significant variables and interactions is presented below:

\begin{align*}&{\rm{LPP} \lt-\rm{lmer}}\left( {{\rm{Voltage} \sim \rm{Sex + Bias + Face + Sex^{*}Bias}}}\right. \nonumber\\ &+ \left.{{{\rm Face^{*}Sex + Face^{*}Bias + Sex^{*}Face^{*}Bias}} + \left( {{\rm{1|Subject}}} \right)\!, {\rm{data}}}\right. \nonumber\\ &= \left.{{{\rm Peaks\_LPP, REML = FALSE}}} \right)\end{align*}

**Fig. 5.**
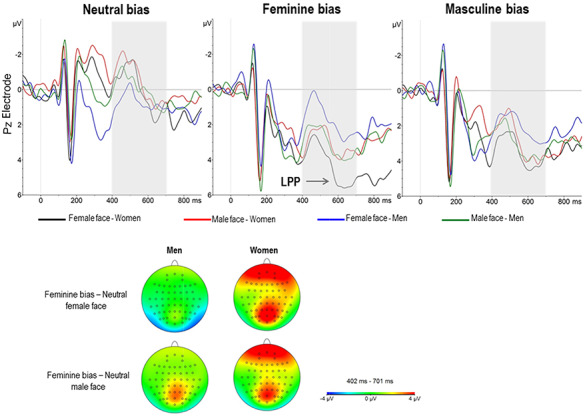
ERP responses elicited by male and female faces in men and women preceded by neutral (left), feminine (center) and masculine (right) sentences. Responses are plotted at PZ electrode. Negative voltage is plotted up (top panel). Voltage scalp distributions (bottom panel).

**Fig. 6.**
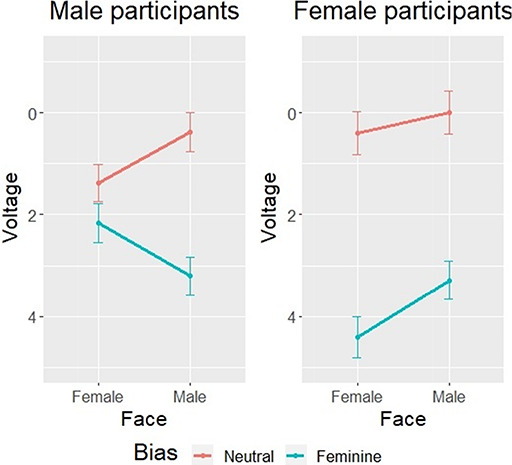
Estimated voltage means in response to male and female faces preceded by neutral and feminine bias, in men and women. Negative voltage is plotted up.

Due to the increasing number of variables, estimates and their corresponding 95% CI of the reduced model are presented in Table 2. As observed, the second-order interaction Sex × Face × Bias was significant. In order to correctly interpret these effects, grand-average ERPs and a mean plot are represented in Figures 5 and 6, respectively.

**Table 2.** Parameter estimates, SEM, corresponding 95% confidence intervals and *P*-values from the linear mixed effects corresponding to the LPP reduced model; *P < 0.05, **P < 0.001

	Estimate	Standard error	95% CI	*P-*value
Intercept	0.39	0.71	[−1.03; 1.81]	0.59
Sex	0.99	1.01	[−1.01; 2.99]	0.32
Feminine bias	4.00	0.53	[2.96; 5.04]	<0.001**
Masculine bias	2.09	0.53	[1.05; 3.14]	<0.001**
Face	−0.38	0.53	[−1.42; 0.67]	0.48
Sex × Feminine bias	−3.21	0.74	[−4.66; − 1.75]	<0.001**
Sex × Masculine bias	0.06	0.74	[−1.40; 1.51]	0.94
Sex:Face	−0.61	0.75	[−2.07; 0.85]	0.41
Sex × Feminine face	−0.73	0.75	[−2.21; 0.75]	0.33
Sex × Masculine face	1.35	0.75	[−0.13; 2.82]	0.07
Sex × Face × Feminine bias	2.77	1.05	[0.71; 4.84]	0.008*
Sex × Face × Masculine bias	−1.34	1.05	[−3.4; 0.73]	0.203

Figure 6 shows that gender-unbiased sentences (neutral) produced similar mean amplitudes for both male and female participants regardless of the face that was presented. However, differences between male and female participants arise when the sentence was biased toward feminine stereotypes. In this sense, two remarkable results were found. First, all participants showed greater LPP amplitudes when these sentences were followed by a male face (i.e. incongruent feminine bias—male face trials) compared with neutrally biased sentences. Second, the most striking result is that female participants showed the largest LPP amplitudes when they were presented a feminine biased sentence followed by a female face (i.e. for the compliance with a female gender stereotype).

## Discussion

The goal of this study was to investigate ERP variations in the processing of facial stimuli as a result of the compliance and violations of gender stereotypes. On this experimental design, the information provided by the context is encoded and integrated with the one provided by the face itself. Thus, gender congruency is processed implicitly with no additional evaluative or gender determination task from the participant.

First, we observed larger N170 amplitudes when facial stimuli were preceded by gender-biased sentences compared with gender-unbiased sentences. Previous studies have found contextual effects on the N170. Most of these studies reported N170 amplitude enhancements with both pictorial and linguistic emotional contexts relative to neutral contexts (Righart and de Gelder, 2006; Ibanez *et al.*, 2010). These findings suggest that the processing of faces at this early stage is modulated by meaningful situational information. As mentioned in the introduction section, some studies have found that directing of attention toward facial stimuli enhances the N170 response (Eimer, 2000; Churches *et al.*, 2010). Accordingly, when participants (males and females) of the present study were provided gender-stereotyped information, the N170 enhancement may be interpreted as an increased attention toward facial features. It could be hypothesized that stereotypes generated an expectation about the subsequent face that would increase the attention to facial features, with the aim of checking whether it corresponded or not to their current expectation. Interestingly, the modulation of the N170 component as a result of the presence or absence of a prior bias (be it feminine or masculine bias) was significantly larger in male participants, compared with female participants. Thus, male participants most likely allocated more attentional resources to process faces when a biased context was presented. This result supports the hypothesis that stereotype activation is higher and maybe harder to suppress in male compared with female participants, in line with results of the study carried out by (Proverbio *et al.*, 2018a). This effect also implies that female participants showed reduced N170 amplitudes compared with men when the face was preceded by a gender-biased sentence. In this sense, the study by Sun *et al.* (2010) found that women contrary to men showed reduced amplitudes of N170 in a gender, relative to an orientation, identification task. The reduced amplitude found in women is interpreted by the authors as a way of processing social information of faces much more efficient, strongly centered on task demands. They propose that this would also explain women’s better performance in face-related tasks (Lewin and Herlitz, 2002; Proverbio *et al.*, 2018b). Beyond the nature of the experimental manipulation, previous studies report a right hemisphere N170 dominance in men (and a more bilateral functioning in women) (Godard *et al.*, 2013) or a bilateral pattern in men and a left lateralized pattern in women (Proverbio *et al.*, 2012). Thus, the literature is still confusing on how solid inter-hemisphere differences between men and women are in N170 as well as on what the direction of the effect is. Despite the present study was not aimed to solve the issue, it reveals yet an additional different pattern of results, adding uncertainty to the issue. Future investigation under different task demands and across several experimental conditions, or even with lateralized visual field stimuli presentations, will shed new light into this issue.

Regarding LPP effects, male participants elicited the largest LPP amplitude in response to male faces that were preceded by female stereotype-biased sentences. This result is in agreement with prior findings of increased N400 amplitudes in men for male agents playing feminine roles in sentences (Proverbio *et al.*, 2018a). Thus, male faces following incongruent gender stereotype-biased contexts were the most salient condition for men compared with the processing of faces that were congruent with their respective gender stereotypes, and with respect to the processing of female faces after male stereotype-biased sentences. According to Moss-Racusin *et al.* (2010), men feel the pressure to stick to their stereotypes, since men not conforming to masculine norms and stereotypes are perceived as weak.

On the contrary, female faces preceded by masculine biased sentences did not elicit a significant LPP response in male participants, which is in line with the fact that women have begun to adopt roles habitually held by men (Diekman and Eagly, 2000; Vandello *et al.*, 2013). Therefore, this result suggests that gender stereotypes are represented in the men participants’ brain, especially the ones concerning them.

Results found for women can be divided in two parts. First, they exhibit similar LPP amplitudes than those found in men participants for male faces preceded by feminine biased sentences. This effect might indicate that gender stereotypes are present not only amongst men, as it was found in the study by (Proverbio *et al.*, 2018a) but also in women. The nature of the stimuli may explain this difference: while Proverbio and colleagues used sentences referring to stereotypes related to sports, jobs and housework, we extended it including personal traits, preferences, behavior and societal expectancies. In this sense, Deaux and Lewis (1984) demonstrated that gender stereotypes vary in their power. For example, trait information is not as linked to a certain gender as physical appearance. Accordingly, our results suggest that some gender stereotypes might be more rooted than others.

Unexpectedly, the largest LPP response in female participants was found for female faces preceded by congruent feminine-biased sentences. Larger positive responses for faces that were emotionally congruent with prior context have previously been found (Aguado *et al.*, 2019a). A higher motivational relevance for this type of condition was claimed to explain this effect. Similarly, larger LPP modulations have been found at the recognition of faces previously tied to negative biographical information about a person (Abdel Rahman, 2011; Kissler and Strehlow, 2017). These studies revealed that contextual information affects post-perceptual evaluative processes in a top-down fashion. In our study, the display of a female face after a feminine-biased sentence was perceived as the most salient event by female participants. A reason that might explain this novel result is the nature of the gender stereotypes that were included in our experiment, related to wider societal expectations from women beyond those of occupation, housework, and sports. According to Hentschel *et al.* (2019), a tendency amongst women has emerged directing them to leave behind their stereotypical frame. The existing asymmetry in the gender stereotypes distribution has changed over the years: people are inclined to think that women’s situation has changed, adapting new roles traditionally held by men and that it will continue to change toward a greater equality and less sex segregation (Diekman and Eagly, 2000). As far as we know, this is the first study presenting an index (i.e. a larger LPP) of a greater difficulty in women to process compliance with a typical female stereotype. Women are probably more aware of gender stereotypes affecting themselves than men. Also, young women report this awareness earlier (Marine and Lewis, 2014). We speculate that these facts might be also responsible for this effect.

Regarding ratings in the Modern Sexism test, there were no differences between male and female participants. This would allow ERP responses to be interpreted as differential implicit processing of gender stereotypes, not necessarily reflected in the participant’s explicit behavioral response. In this sense, self-reported attitude toward sexism did not account for variability in amplitudes of the components examined. Results on this test cannot be directly linked to our electrophysiological data. Items on this test were mainly focused on the existing subtle sexism toward women nowadays, but significant differences in the ERPs point in a different direction. Large LPP amplitudes in both men and women participants for male agents in feminine roles cannot be correlated to the scores on this test, since men’s situation in current society was not measured. Nevertheless, the largest LPP response was elicited when female faces were preceded by feminine biased sentences only in women participants. Based on the lack of significant differences between men and women’s scores on this test, we might have expected the same pattern in male participants. The degree of self-identification with a woman or a man agent, as suggested by (Proverbio *et al.*, 2018a), may explain this asymmetry.

The present study used a sample of young participants (aged between 18 and 35 years). Further studies are needed to determine whether participants of older generations would reveal dissimilar results.

In sum, the present data shed new light onto the online unfolding of social factors (i.e. gender stereotyping) upon face processing. The findings of the present study have important implications for understanding the differential assumptions of males and females about gender roles. Brain responses, which are not subject to conscious control, reveal a differential impact of processing a human face after a statement has been stereotypically biased to the expectation of a male or a female. First, the processing of facial stimuli at very early stages (N170) is modulated by contextual gender-stereotyped information, especially in the case of male participants. Second, males assuming a role typically associated to females still causes cognitive conflict, as reflected in the LPP component. Finally, the fact that females showed larger LPP amplitudes when a female face was linked to a traditional female-stereotyped statement can be interpreted as the most conflicting or salient event for them to process, which might be tied to some short of struggling to accept compliance with traditionally held female roles. Overall, the examination of automatic brain responses that are beyond conscious control allows us to conclude that gender stereotypes are still deeply rooted in our culture and society, with male and female individuals reacting differently to them.
